# Maternal and newborn health implementation research: programme outcomes, pathways of change and partnerships for equitable health systems in Uganda

**DOI:** 10.1080/16549716.2017.1359924

**Published:** 2017-09-06

**Authors:** Asha George, Moses Tetui, George W Pariyo, Stefan S Peterson

**Affiliations:** ^a^ School of Public Health, University of the Western Cape, Bellville, Republic of South Africa; ^b^ Department of International Health, Johns Hopkins Bloomberg School of Public Health, Johns Hopkins University, Baltimore, MD, USA; ^c^ Makerere University School of Public Health (MakSPH), Makerere University, Kampala, Uganda; ^d^ Epidemiology and Global Health Unit, Department of Public Health and Clinical Medicine, Umeå University, Umeå, Sweden; ^e^ Health Section, Programme Division, UNICEF NY, New York, NY, USA; ^f^ International Maternal and Child Health, Department of Women’s and Children’s Health, Uppsala University, Uppsala, Sweden

Nestled in eastern and southern Africa, with a population of just over 39 million, Uganda reported a maternal mortality ratio of 360 per 100,000 live births, a neonatal mortality rate of 19 per 1000 live births and an infant mortality of 38 per 1000 live births in 2016 [1,2]. While health outcomes have been improving, much more progress could be realized, particularly given that the majority of maternal and child health deaths are avoidable. Contact with the health system occurs, but not when it is most needed. In Uganda, although 95% of mothers received antenatal care from a skilled provider for their most recent live birth, only 59% of live births in the past 5 years were delivered by a doctor or nurse/midwife, and 57% were delivered in a health facility in 2011 [3].

Progress is also constrained by barriers to accessing healthcare for women and children that interact across health-system levels and increase across the continuum of care [4–6]. Once the need for healthcare seeking is recognized, lack of preparedness to mobilize transport, funds, family and community support can delay access to care. Even after family members mobilize their resources to reach a health provider, the poor quality of care provided can still negate the efforts made to seek care in the first place. These penalties of low awareness and low preparedness on the demand side combine with poor access and poor quality of care on the supply side, disproportionately prejudicing those who are most marginalized.

The dynamics of inequality that characterize maternal and child health in Uganda have multiple dimensions. The percentage of births delivered by a skilled provider is substantially higher in urban areas (90%) than in rural areas (54%) [3]. Coverage of maternal and child health interventions also varies by geographic region, from 57% to 70% [7]. Although these geographic inequalities are decreasing, income inequality in maternal healthcare is increasing [8]. Social norms that replicate inequalities related to gender, age and marital status, among other forms of social hierarchy, also debilitate maternal and child health.

Rather than being daunted by these substantial and varied barriers, a team at the Makerere University School of Public Health, Kampala, embraced the importance of a systems lens that considers how numerous levels and partners dynamically interact to advance or stall maternal and newborn health [9]. They built on previous interventions that led to significant gains in community newborn health practices and facility deliveries [10]. The extent to which these single interventions sustained health gains was partly limited by their isolated implementation within the dynamic, broader health systems in which they were embedded [9]. Knowing that they had to address capacity gaps across health-system levels, the team sought to consolidate the gains previously realized by embarking on the Maternal and Neonatal Implementation for Equitable Systems project (MANIFEST) detailed in this volume [11].

MANIFEST supported community outreach in the form of community health worker (CHW) home visits, coupled with community awareness (dialogues, radio) and community capacity strengthening (community savings and transport initiatives) to ensure that, with community support, mothers and families felt empowered to seek timely care [12,13]. To ensure that this increased demand was met with quality services, MANIFEST also facilitated enabling environments for healthcare workers to respond through supportive supervision, clinical mentoring and participatory action research steered by local district health teams [9,14,15].

## Programme outcomes

Through these multi-layered initiatives, the MANIFEST project demonstrated significant improvements in early antenatal care attendance, facility deliveries and newborn care practices [9,16]. Kananura et al. also demonstrated significant improvements from baseline levels in birth preparedness and knowledge of obstetric danger signs in rural communities [16]. The increases in knowledge of at least two newborn danger signs and at least three obstetric danger signs are higher than those achieved in other parts of the country [17,18].

While improvements in newborn care practices were statistically significant, the absolute changes in kangaroo mother care for low-birth-weight newborns and delayed bathing for all babies were minimal. Social norms and hierarchies that inhibit care seeking remain a challenge. Younger women aged 15–24 years compared to those aged 35 years and above were associated with reduced odds of birth preparedness given knowledge of obstetric danger signs [16]. Changing the behavioural practices embedded in sociocultural contexts requires time and engagement with multiple stakeholders at both family and community level [19].

Another concern was that some of the improvements seen were not significantly higher than those in comparison areas, as other implementing partners were promoting similar safe motherhood initiatives in comparison areas. The lack of virgin comparison areas when implementing effective interventions challenges evaluators to pursue other evaluation metrics and questions that examine not just programme outcomes [20], but also pathways of change and partnerships formed. This editorial seeks to highlight some of those lessons related to critical pathways and partnerships based on the papers included in this special issue.

## Pathways of change

### Catalysing health workers to improve provision of quality services

MANIFEST-supported health workers improve service provision through a system of cascade mentoring, with external specialists mentoring teams of local mentors [15]. Key improvements in increased productivity, improved management of patients, increased problem identification and solving, and improved health worker skills were reported. Yet implementation was not straightforward. Documentation of the mentorship process through mentees’ self-assessment tools was unpopular, and these tools were not used owing to their being perceived as burdensome and to fears about assessment. Despite careful selection, several local mentors dropped out and only those who were passionate about maternal and newborn care remained. Higher level cadres were less likely to be consistent mentors owing to their own workload and poor attitude towards mentorship. Nonetheless, key supportive elements included the role of district leadership and the initial involvement of external mentors. Overall, Ajeani et al. [15] indicate that local staff with the right aptitude, with training and mentoring themselves, can support frontline health workers to improve service delivery through mentoring, provided that facilitation in terms of transportation and compensation of time/work demands can be addressed. Emerging research on mentoring in other low- and middle-income contexts indicates cost-effective improvements in facility readiness, nurse knowledge and adherence to protocols, as shown by case sheets [21].

In addition to mentoring, MANIFEST strengthened quarterly supportive supervision focused on maternity care services and key system inputs to ensure quality provision of such care [14]. Supervisors and health workers jointly identified opportunities for improvement through observation and review of health-facility data at each of the facilities. Joint problem solving and follow-up of action points were undertaken in a participatory manner, resulting in improvements in laboratories, basic pharmacy, lighting in the maternity and delivery wards, infection control, waste segregation and placenta pits. Engagement by district managers enabled in the process facilitated support from other system partners and levels. Nonetheless, Kisayke et al. [14] also found that some elements of service quality, such as referral readiness and running water in facilities, were harder to achieve. While the engagement of midwives improved, the majority of the doctors did not participate in the mentorship sessions. Iterative team engagement with data to identify problems and track progress on implementing local solutions is a hallmark of quality improvement cycles [22], but they also need to address organizational culture and norms that may inhibit frontline health workers’ critical engagement and ability to implement the required changes [22,23].

In examining the organizational factors that motivated health workers to stay in rural areas, Kiwanuka et al. [24] found that these health workers accepted their work environment with an attitude of resignation and rationalized that working in a difficult environment proved their mettle. Improved infrastructure was not seen as a motivator, possibly as all rural districts were uniformly bad. Incentives also were minimally valued, as they rarely materialized despite being mandated. While staff accommodation reduced travel expenses and living costs and kept families together, health workers highlighted the advantage of staying far away from health facilities as a strategy to avoid excessive workloads. Despite these difficult working conditions, they valued working for the public sector, given the comparative lack of job security in the private sector. Elements of vocation, the need to be near family, maintaining community ties and having opportunities to invest locally kept health workers in rural districts. Retaining health workers in rural areas requires bundled interventions that address both intrinsic and extrinsic motivation linked to living environments, working conditions and development opportunities tailored to local contexts [25,26].

### Strengthening CHWs

MANIFEST raised awareness of maternal and newborn health at community level through a variety of communication channels, including CHWs and village health teams (VHTs), community dialogue meetings, radio talk shows and health facility workers. Kananura et al. [16] found that knowledge of obstetric danger signs and facility delivery seeking were higher among intervention residents who were visited by the VHTs. This is triangulated by another finding from the study that found that VHTs were the highest source of maternal and newborn health information at community level. Nonetheless, 1 year after VHTs were trained in maternal and newborn health, Namazzi et al. [12] found that while VHT knowledge on maternal danger signs remained stable, at over 70%, their knowledge of newborn danger signs and care practices declined, from 85.5% to 58.9% and from 75.3% to 50.3%, respectively.

Considering the costs of facility supervision and retraining, MANIFEST supported peer supervision of VHTs and found that it was cheaper and more reliable as VHTs were able to relate to and learn better from their peers. Research on CHWs in other contexts has also supported innovations in CHW supervision going beyond their facility-based supervisors [27–29]. MANIFEST also found that non-monetary incentives such as T-shirts, certificates and *musawo* (doctor) status in the community motivated VHTs to continue working. At the same time, monetary incentives and means of transport such as bicycles were found to be essential for VHTs to undertake their roles. Other studies also indicate a nuanced approach to CHW motivation requiring both financial and non-financial incentives [30–32].

Despite being such a resource, supporting CHWs requires ongoing efforts and entails addressing health system challenges related to the training, supervision and motivation of this important workforce [33]. Over the past 10 years there has been a seven-fold increase in low- and middle-income country CHW publications, driven largely by vertical programmes [34]. While CHWs are now recognized as vital actors in realizing health outcomes, issues of sustainability linked to supervision, motivation and performance indicate that initiatives need to move beyond specific programmes and address how CHWs serve and are supported by the communities and health systems to which they belong [35].

### Enabling community savings and transport groups

In addition to CHWs, MANIFEST sought other ways of building community capacity to support their role in improving maternal and child health outcomes. Mutebi et al. found that despite widespread poverty, there were many savings groups, albeit not necessarily for health [36]. Yet savings groups for emergency illness and death presented an opportunity to include savings for maternal and newborn health. In addition, some savings groups supported educational and sensitization activities, providing an opportunity to educate members about health. Several reviews on microfinance indicate its important effects on health awareness and care seeking. While some reviews affirm impacts on health outcomes, others are more cautious, suggesting the need for further longitudinal research to evaluate impact pathways and effects [37–39].

In Uganda, MANIFEST found that many of the savings groups assessed were in need of strengthening [36]. Most were informally keeping money in a safe box or with a treasurer, leaving them prone to mismanagement, with few using innovations linking them to banking via mobile phones. Challenges faced by savings group members included failure to recover loans from members, lack of training in financial management, irregular group meeting attendance, registration challenges, and high illiteracy levels among members.

In evaluating their experience of supporting community savings for maternal and newborn health, Ekirapa-Kiracho et al. found that families saved more for health and that having saved money was a predictor for facility delivery [13]. There was a 6% increase in the proportion of women who saved for health through savings groups in the intervention compared to the control; however, the majority seemed to have saved informally and on their own. Although the savings groups seem to hold potential to improve saving for health, more evidence about their effectiveness in comparison to other informal saving methods is required. Exclusionary practices such as having a fixed monthly savings target and age criteria for membership had to be revised, particularly since they discriminated against the young and the poorest, who were already at risk for poor maternal and newborn health outcomes. Another key lesson was the need to include husbands in sensitization activities since they are the key decision-makers, particularly with household finances. Finally, MANIFEST also realized that local community development officers were not able to support the savings groups owing to their lack of transport funds and competing responsibilities. More partnerships with other local existing government staff and non-governmental organizations are therefore required to provide the much-needed support to savings groups.

While the project initially prioritized identifying transport before delivery, because of the availability of drivers, particularly during the day, women tended not to secure transport in advance. It appears that having money was more crucial for securing transport once it was needed [13,36]. The benefits of having preidentified transport was more critical at night, when it is generally difficult to get transport. In Tanzania, CHWs and community financing of transport services for maternal health continued to be sustained beyond the original project timelines. Given the rising recognition of multi-sectoral action in improving health, these lessons on how to work with savings and transport groups have direct relevance for the Sustainable Development Goals [40,41].

## Partnerships

In contrast to previous efforts, MANIFEST not only sought to work with multiple health systems levels and actors concurrently, but did so by enabling local partners to lead in implementation and learning. Collaboration in this manner enabled the testing of ideas during iterative cycles of action, review of data and reflection on experiences [42]. The pathways of change were not uniformly successful and at times they revealed new facets of implementation.

In identifying health worker mentor candidates, criteria linked to relational skills, trust and commitment were more important than formal qualifications and professional hierarchy. Similarly, supervision and mentorship also yielded improvements, despite uneven participation across professional hierarchies. The partnerships forged with district-level teams and external mentors were critical in providing additional support to these supply-side initiatives.

On the demand side, while awareness of certain health conditions improved, this did not stay constant even among CHWs. Health behaviours that entailed changing social norms required further support and engagement with a broader range of cultural brokers. Similarly, partnerships with community savings groups and transporters also led to new learning. Savings groups required significant capacity building and reform to ensure the inclusion and trust essential for handing over savings by those most marginalized. Men emerged as a key stakeholder in changing savings for maternal health and in supporting care seeking. Transportation needs and preparedness strategies needed to be adapted to time of day. VHTs were a critical resource supporting these varied community relationships. At the same time, MANIFEST showed clearly that VHTs also require further community and health system support to sustain their contributions.

The lessons learned from working closely together to address these implementation experiences as they emerged played an important role in invigorating stakeholders across health systems levels and implementation structures. At the same time, stakeholders faced risks in welcoming constructive yet challenging dialogue about their roles in improving maternal health outcomes within the local health system. Differing points of view emerged between key stakeholders, including between Makerere researchers and the district health teams. The researchers were concerned with the ambiguity of boundaries among stakeholders and the lack of clear delineation of responsibility, yet the health managers felt too closely monitored by the researchers. Furthermore, facilitating wide stakeholder involvement entailed conflict, stress and uncertainty. Through this process of transformative learning to improve and sustain maternal health outcomes, tolerance, collaboration and being risk conscious emerged as critical values and skills for the partnerships involved [42].

A central ethos underpinning MANIFEST was the participatory manner in which it supported stakeholder engagement, local ownership and responsibility. As the papers in the special issue indicate, this was not without risks and unanticipated implementation outcomes. However, the learning and relationships generated form a critical basis for sustaining progress. Makerere colleagues and the MANIFEST project are at the forefront of current trends that emphasize the importance of embedded implementation research. In this way, they strengthened inclusive partnerships and supported varied pathways tailored to catalyse and transform context-specific programmatic learning into sustained health outcomes [43,44].

## Paper context

This is an editorial that provides an overview of the MANIFEST special issue. The special issue covers 10 papers whose main purpose is to share findings from a maternal and neonatal health intervention in Uganda. The MANIFEST intervention used a participatory action research approach to engage different actors to improve maternal and neonatal health outcomes in the districts of Pallisa, Kibuku and Kamuli, in eastern Uganda.
